# Genome-Wide Transcriptomic Analysis of *n*-Caproic Acid Production in *Ruminococcaceae* Bacterium CPB6 with Lactate Supplementation

**DOI:** 10.4014/jmb.2107.07009

**Published:** 2021-08-27

**Authors:** Shaowen Lu, Hong Jin, Yi Wang, Yong Tao

**Affiliations:** 1 CAS Key Laboratory of Environmental and Applied Microbiology and Environmental Microbiology Key Laboratory of Sichuan Province, Chengdu Institute of Biology, Chinese Academy of Sciences, Chengdu 610041, P.R. China; 2 School of Basic Medical Science, Chengdu Medical College, Chengdu 610083, P.R. China; 3 Faculty of Bioengineering, Sichuan University of Science and Engineering, Xueyuan Street 180#, Huixing Rd. Zigong 643000, P.R. China; 4 Department of Biosystems Engineering, Auburn University, Auburn, Alabama, Alabama 36849, USA

**Keywords:** *n*-Caproic acid, lactate, chain elongation, transcriptome, RNA-Seq

## Abstract

*n*-Caproic acid (CA) is gaining increased attention due to its high value as a chemical feedstock. *Ruminococcaceae* bacterium strain CPB6 is an anaerobic mesophilic bacterium that is highly prolific in its ability to perform chain elongation of lactate to CA. However, little is known about the genome-wide transcriptional analysis of strain CPB6 for CA production triggered by the supplementation of exogenous lactate. In this study, cultivation of strain CPB6 was carried out in the absence and presence of lactate. Transcriptional profiles were analyzed using RNA-seq, and differentially expressed genes (DEGs) between the lactate-supplemented cells and control cells without lactate were analyzed. The results showed that lactate supplementation led to earlier CA p,roduction, and higher final CA titer and productivity. 295 genes were substrate and/or growth dependent, and these genes cover crucial functional categories. Specifically, 5 genes responsible for the reverse β-oxidation pathway, 11 genes encoding ATP-binding cassette (ABC) transporters, 6 genes encoding substrate-binding protein (SBP), and 4 genes encoding phosphotransferase system (PTS) transporters were strikingly upregulated in response to the addition of lactate. These genes would be candidates for future studies aiming at understanding the regulatory mechanism of lactate conversion into CA, as well as for the improvement of CA production in strain CPB6. The findings presented herein reveal unique insights into the biomolecular effect of lactate on CA production at the transcriptional level.

## Introduction

The increasing demand for fuels and chemicals, and the scarcity of fossil resources necessitate the development of sustainable and innovative strategies for the industrial production. *n*-Caproic acid (CA) has recently gained considerable attention due to its high value as a chemical feedstock [1]. Organic residual streams (*e.g.*, food waste and brewery wastewater) have great potential to be employed as feedstock for CA production [2, 3]. Many studies show that the addition of ethanol during the acidification of wastes can promote chain elongation, and lead to higher volumetric production rate and a high CA selectivity [4, 5]. Generally, biosynthesis of CA is achieved by some anaerobic microbes via the reverse β-oxidation pathway with ethanol as electron donor (ED) [4, 6], in which the oxidation of ethanol provides energy and acetyl-CoA for the chain elongation [7]. In addition to ethanol, many chemicals are explored as EDs for CA production, including hydrogen [8], methanol [9], propanol [10], and *D*-galactitol [11].

Recently, lactate is becoming a potential alternative to ethanol for the production of CA [12, 13]. Lactate can be efficiently converted into CA by a *Ruminococcaceae* bacterium CPB6 [14]. The phylogenic analysis based on 16S rRNA sequences and the whole genome show that strain CPB6 might belong to a new clade (genus) of the family *Ruminococcaceae*, it is thus tentatively christened with the name *Ruminococcaceae* bacterium CPB6 [14]. Strain CPB6 can produce CA (C6) from lactate (as ED) with C2-C4 carboxylic acids as electron acceptors (EAs), or heptoic acid (C7) from lactate with C3-C5 carboxylic acids [15]. More recently, complete genomic sequencing and annotation show that strain CPB6 encodes most genes related to glycolysis and the reverse β-oxidation pathway [16]. However, to date, very little information is available on genome-wide transcriptomic analysis of strain CPB6 for CA production using lactate as ED, which is essential to understand the effect of lactate on the metabolic pathway shift for CA production at the molecular level, and thus elucidate proper strategies for further strain improvement.

RNA-sequencing (RNA-Seq) is a powerful technique to investigate entire transcriptomes, and identify specific genes for the particular interesting metabolic pathways [17, 18]. In this study, RNA-Seq of the transcriptome of strain CPB6 was carried out to investigate the effect of lactate supplementation on gene expression, as well as to identify key genes related to CA production. These candidate genes are likely to be valuable for the metabolic engineering in the future to further improve the ability of strain CPB6 to convert lactate into CA.

## Materials and Methods

### Microorganisms, Media and Fermentation Experiment

*Ruminococcaceae* bacterium CPB6 (GDMCC No.60133) is a spore-forming, obligate anaerobic bacterium that can produce CA from lactate [14]. Strain CPB6 was routinely cultured at 37°C anaerobically in a modified tryptone-glucose-yeast extract (mTGY) medium containing the following compounds (pH 6.0): 5.0 g/l tryptone, 2.0 g/l glucose, 3.5 g/l sodium acetate, 0.41 g/l K_2_HPO_4_·3H_2_O, 0.23 g/l KH_2_PO_4_, 0.25 g/l NH_4_Cl, 0.20 g/l MgSO_4_·7H_2_O, 2.5 g/l NaHCO_3_, 0.50 g/l L-cysteine, 0.25 g/l Na_2_S·9H_2_O, 0.0005 g/l resazurin, and 1 ml of trace element solution SL-10 and 1ml of vitamin solution [13]. The suspension of activated strain CPB6 was inoculated with a 5% ratio into the same medium as described above, and incubated for 12 h until the optical density at 600 nm (OD_600_) of the culture reached 0.8-1.0. Then the culture would be used as the seed inoculum (5% ratio, v/v) for batch experiments. To investigate the effect of lactate on cell growth and CA production, 5 g/l sodium lactate was supplemented into the mTGY liquid medium (*i.e.*, mTGYL). Batch experiments were performed in 250 ml serum bottles containing 100 ml of mTGY or mTGYL media. The headspace of the bottle was filled with highly pure N_2_. Each fermentation was performed in triplicate. The fermentation was carried out at 37°C in an E500 anaerobic workstation (Gene Science, USA) under N_2_: CO_2_: H_2_ (volume ratio of 80:10:10) atmosphere.

Samples were taken at specific times and processed for cell concentration determination and high-performance liquid chromatography (HPLC) analysis. Samples for RNA isolation were taken at the cell growth and stationary phases.

### Analytical Methods

Culture growth was monitored by measuring the OD_600_ using a TU-1810 UV/Vis Spectrophotometer (Puxi Instrument Co. Ltd., China). Lactic acid, acetic acid, butyric acid, and caproic acid were quantified using an HPLC system (Agilent 1260 Infinity, USA) equipped with a differential refraction detector (RID) and a Agilent Hi-Plex H column (300 × 6.5 mm) following the procedure as previously described [15].

### RNA Isolation, Library Construction, and Sequencing

In preparation for RNA isolation, 10 ml cell culture was harvested at each time point, and centrifuged at 8,000 ×*g* for 10 min at 4°C. Cells were then frozen in liquid nitrogen prior to storage at -80°C. The RNA was extracted and purified using a RNA extraction kit (DP430, Tiangen Biotech, China) following the manufacturer's protocol. RNA quality and quantity were characterized using a NanoDrop2000 (NanoDrop Technologies, USA), agarose gel electrophoresis (RNA integrity detection) and Agilent 2100 (RIN value measurement). Only the RNA samples with high-quality (≥ 5 μg; ≥ 200 ng/μl; OD260/280=1.8~2.2; RIN > 6.0) were used for the cDNA library construction and sequencing. Before library construction, rRNAs were removed with the Ribo-Zero rRNA Removal Kit (Epicentre, USA) following the manufacturer’s protocol. The enriched mRNA was randomly fragmented into 200 bp fragments by fragmentation buffer. The mRNA was then the first strand cDNA was synthesized using the random hexamer-primer with the mRNA fragment as the template. After synthesizing the second strand cDNA using DNA polymerase I and RNase H, double-stranded cDNA was further end repaired, A-tailed, and indexed adapters ligated. The final cDNA library was constructed using TruSeq RNA sample preparation Kit (Illumina Inc., USA), and then sequenced by Illumin Hiseq 4000 (Illumina Inc.) with 2 × 150 bp.

Sequencing trimming and quality control methods are as follows: (1) Remove the Adapter sequence in reads;(2) The bases containing non-A, G, C and T at the 5 'end were removed by shear; (3) Trim the ends of reads with low sequencing quality (< Q20); (4) Reads containing 10 % N were removed; (5) Remove Adapter and small segments with length less than 25 bp after quality pruning.

### RNA-Seq Data Analysis

Raw data were processed, and reads containing adapter and poly-N sequences and low-quality reads were removed using Sickle and SeqPrep to obtain clean data [19, 20]. The trimmed reads in each sample were aligned to strain CPB6 genome (CP020705.1) using Bowtie2, and those that did not align uniquely to the genome were discarded using the default quality parameters [21]. Each base was assigned a value based on the number of mapped sequence coverage. Gene expression levels were defined using the number of transcripts per million (TPM), which is proportional to the quantity of cDNA fragments derived from the gene transcripts. The quantitative gene expression values between samples were identified by calculating the number of unambiguous tags for each gene and then normalizing this to TPM, which was calculated following the method reported by Parto *et al*. [22]. The gene expression results were visualized as a heat-map plot using ggplot2 package. The general changes in gene expression among different treatments were evaluated by permutational multivariate analysis of variance using the function Adonis in the R vegan package. Gene annotation was performed based on the Kyoto Encyclopedia of Genes and Genomes (KEGG) database (http://www.genome.jp/kegg/).

### Reverse Transcription-Quantitative PCR (RT-qPCR)

In order to validate the results of the RNA-Seq analysis, 5 candidate reference genes were selected for RT-qPCR confirmation. Primers used are listed in Supporting Information Table S3. Total RNA was extracted from three sets of independent cultures grown on cultures with or without lactate supplementation, and then converted to cDNA by random priming, using the Maxima Reverse Transcriptase (Thermo Scientific). PCR reactions were run in triplicate using procedure as follows: initial denaturation (3 min at 95°C), followed by 45 cycles of denaturation (5 s at 95°C), annealing and elongation (30 s at 60°C). The transcription level of genes was determined according to the 2^-(ΔΔCt)^ method, using 16S rRNA as a reference gene for the normalization of gene expression levels [23].

### Statistical Analysis

Significant differences of the gene expression between the culture with lactate supplementation and the control were determined using ANOVA in R software (version 3.5.2). TPM values were first transformed to log10-scale. The log10-transformed TPM values were then properly centered for better representation of the data using the heatmap plots. Fold changes (FCs) as the ratio of the TPM values were calculated following the method reported by Love *et al*. [24], and were used to compare the differentially expressed genes (DEGs) between the culture from fermentation with lactate supplementation and the control.

### RNA-Seq Data

The RNA-Seq sequencing data have been deposited in the NCBI Sequence Read Archive (SRA) under the accession number PRJNA564589

## Results

### Cell Growth and the Production of CA

**Fig. 1:**
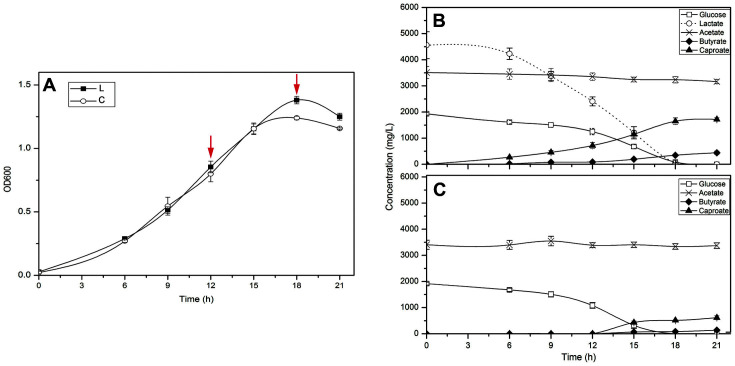
Fermentation kinetics of *Ruminococcaceae* bacterium CPB6. (**A**) Cell growth profiles. Time points for taking samples subjected to RNA-Seq are indicated with red vertical arrows. L: fermentation with lactate supplementation; C: control fermentation without lactate supplementation; (**B**) Sugar consumption and metabolites production during the fermentation with the supplementation of lactate; (**C**) Sugar consumption and metabolites production during the control fermentation. Values represent the mean of the biological triplicates and error bars represent the standard deviations.

As shown in Fig. 1A, cells took approximately 18 h to grow to the stationary phase. Although the maximum OD_600_ of the lactate-supplemented cultures was slightly higher than that of control cultures without lactate at the stationary phase (1.25 vs 1.16), both cultures showed similar growth kinetics. The lactate was completely consumed in the lactate supplemented culture after 21 h of cultivation. Moreover, no lactate was detected throughout the control group. CA production was started to be observed in the lactate-supplemented cultures after 6 h of cultivation, and the CA titer continued to increase and reached 1,717.2 mg/l at 21 h (Fig. 1B), while CA was not detected in control cells until 15 h of cultivation, and the CA titer of which only reached 618 mg/l at 21 h (Fig. 1C). These results suggest that lactate supplementation had little effect on the cell growth, but led to earlier initiation for CA production (6 vs 15 h), higher final CA titer (1,717 vs 618 mg/l), and higher CA productivity (81.8 vs 29.4 mg/l h).

### RNA-Seq Statistics

Samples were taken for RNA-Seq analysis from both growth (12 h) and stationary (18 h) phases for the lactate-supplemented cultures and control cultures. For each culture, independent biological triplicates (a, b, and c) were included (Table 1). Therefore, a total of twelve samples were taken for cDNA libraries construction and sequencing on the Illumina HiSeq 4000 (Illumina). The number of raw reads generated from the sequencing for each library was from 15.7 to 23.5 million (Table S1). A total of 224 Mb sequence reads from 12 cDNA libraries were mapped to strain CPB6 genome. Only those reads that mapped unambiguously to strain CPB6 genome were used for further analysis.

Overall, out of the reads derived from all samples, 15.1 to 22.7 million reads were unambiguously mapped to strain CPB6 genome, and over 98% reads were mapped (Table 1). A total of 1968/1969 out of 2045 protein-coding genes had detectable expression in all cells, covering 96% of strain CPB6 genome. This result indicated that the RNA-Seq analysis achieved comprehensive coverage of strain CPB6 transcriptome. The transcription levels (the number of transcripts per million, TPM) of most active protein-coding genes were in the range of 3.2 × 10^4^ –7.3×10^4^.

**Fig. 2:**
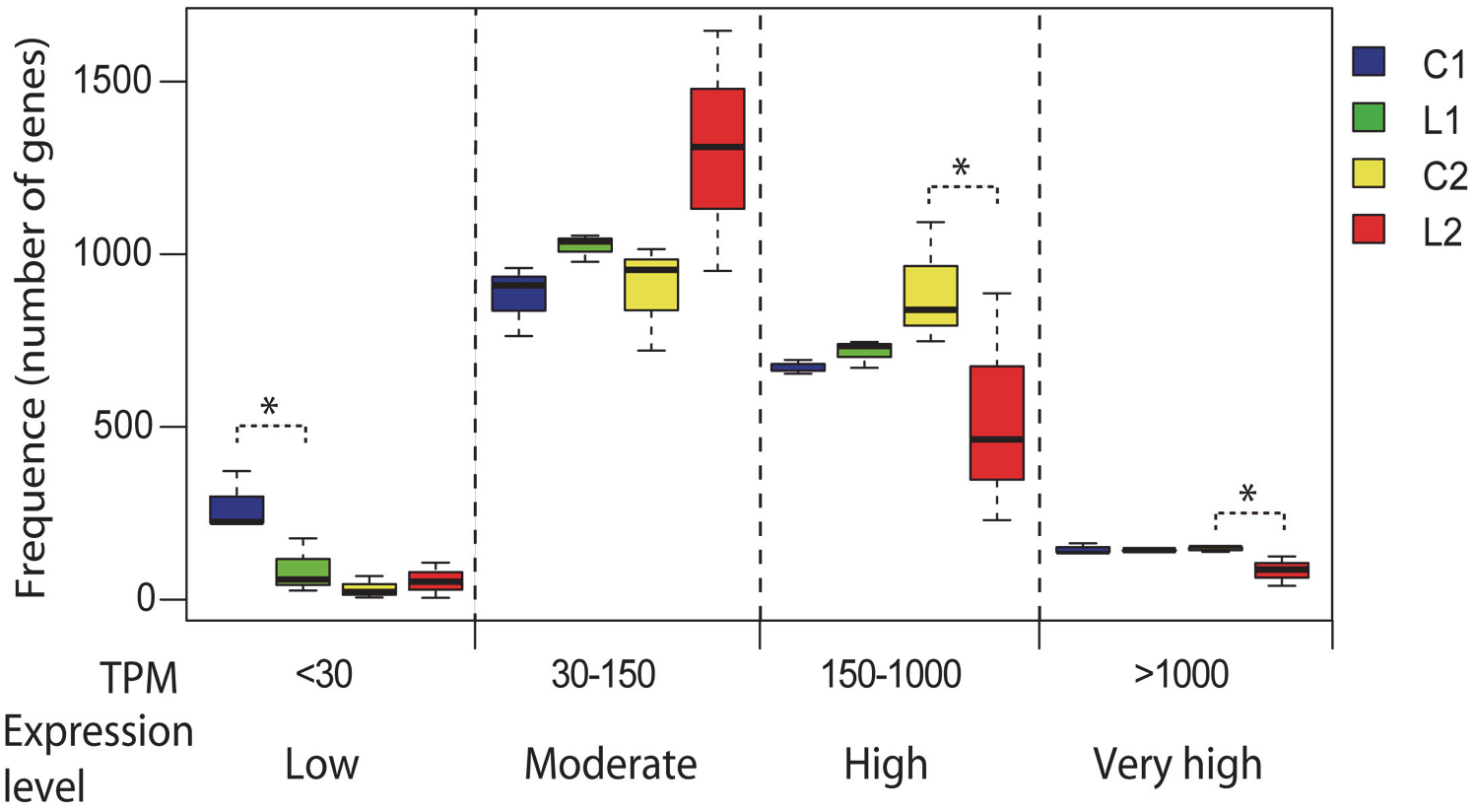
Frequency histogram of transcripts from the RNA-Seq results. L: the lactate supplemented cells; C: control cells without lactate supplementation; Number 1 and 2 represented the growth phase and stationary phase, respectively. The diagram shows the distribution of the number of genes expressed at different transcripts per million (TPM) levels. The percentage value above each bar indicates the genes at the specific expression level accounting for the proportion of the total number of genes. The ‘*’ mark indicates that significantly different frequencies (*i.e.*, numbers of genes) were observed between the two RNA-Seq data sets from the lactate-supplemented fermentation (L) vs. the control (**C**), respectively.

As illustrated in Fig. 2, the gene expression could be classified into four levels: low (TPM < 30), moderate (TPM: 30-150), high (TPM: 150-1000), and very high (TPM > 1000). The number of genes at some specific expression levels was significantly different for the two cultures. For the growth phase, there were slightly more genes in the moderate, high and very high expression level in the lactate-treated cells than in control cells, but lowly expressed genes were significantly decreased. While for the stationary phase, the lactate-treated cells had more genes in the moderate expression level, but fewer genes in the high and very high expression level.

### Functional Annotation and Classification

**Fig. 3:**
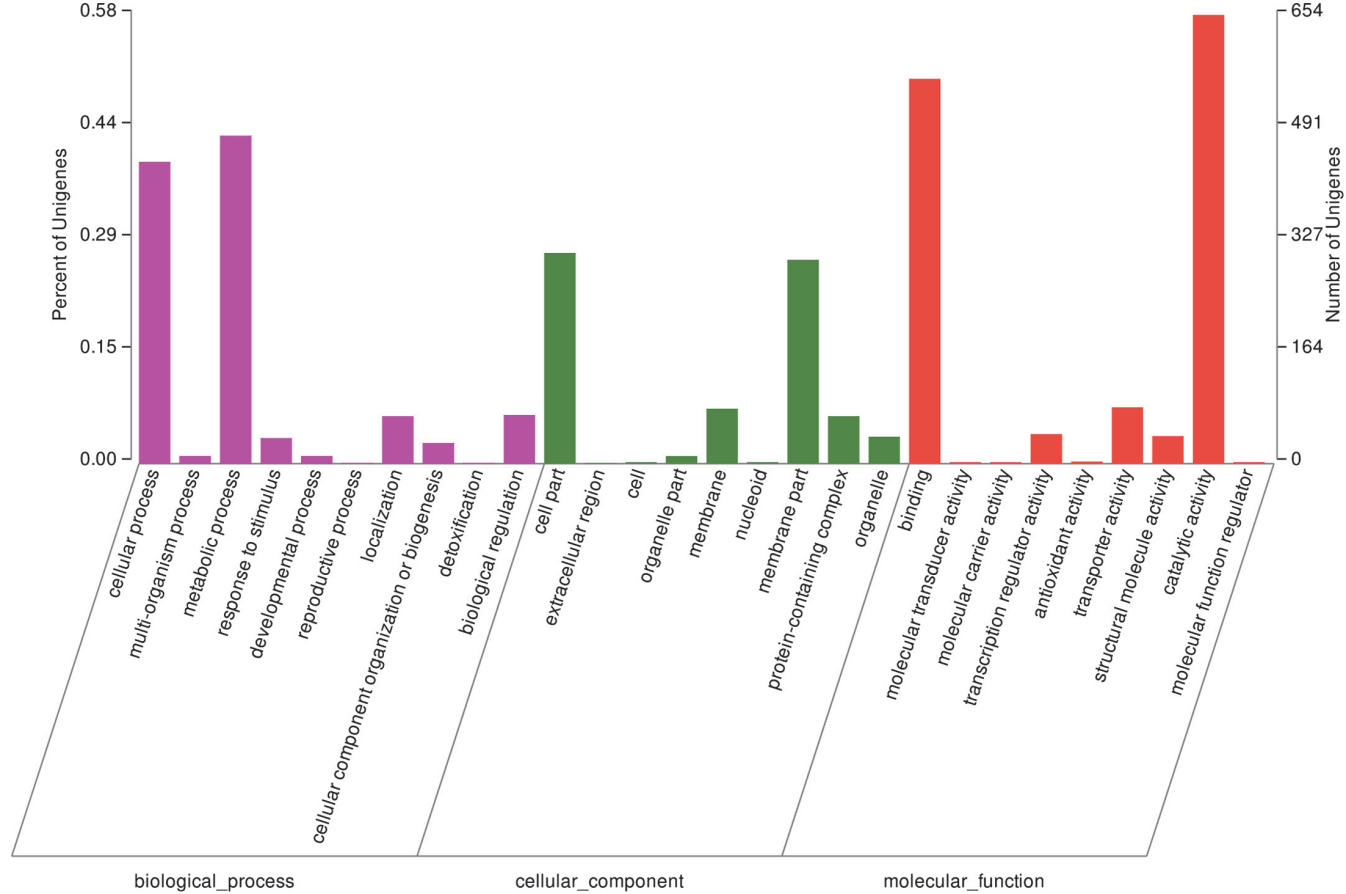
Annotation of genes using Gene Ontology (GO) in the transcriptome of strain CPB6. Left axis: the proportion of genes falling into each GO category; right axis: the number of genes falling into each GO category.

In the transcriptome of strain CPB6, a total of 1122 expressed genes were allocated into three primary Gene Ontology (GO) categories (Fig. 3), including the category of biological process (601 genes), cellular component (524 genes), and molecular function (916 genes). In each category, the genes were further assigned into 28 functional groups, such as metabolic process (478 genes), cellular process (440 genes), cell part (307 genes), membrane part (297 genes), catalytic activity (654 genes), binding (561genes), and etc. The analysis of the genes based on the KEGG annotation identified a total of 1046 unigenes allocated into six primary KEGG categories including 35 subcategories (Fig. S1). The analysis based on the Clusters of Orthologous Groups (COGs) showed that 1785 unigenes were allocated to four primary COG categories containing 20 COG functional clusters (Fig. S2).

### DEGs Affected by Lactate Supplementation

The correct identification of DEGs between specific conditions is a key in understanding phenotypic variation of organisms under environmental stress. As shown in Table 2, only 34 DEGs (FC ≥2 or ≤ 0.5 with p-value < 0.05) were found in the lactate-supplemented cells compared to control cells at the growth phase, of which 15 genes were upregulated, and 19 genes were downregulated. At the stationary phase, a total of 245 DEGs were identified in both cultures, of which 123 genes were significantly upregulated and 122 genes were downregulated (Table S2). These results demonstrated that the addition of lactate led to differences in gene expression during different growth phases.

The COG distribution of DEGs is illustrated in Fig. S3. It revealed potential genes, processes and pathways that may participate in the utilization of lactate and CA production. Cluster analysis of the DEGs between the lactate-supplemented cells and control cells is showed in Fig. S4. Obviously, more DEGs was observed in the stationary phase (L2 vs C2) than in the growth phase (L1 vs C1).

**Fig. 4:**
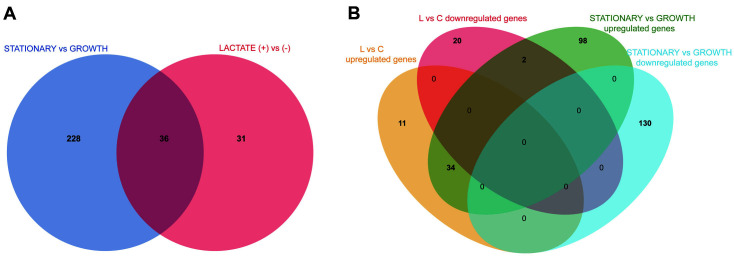
Venn diagram of the numbers of differentially expressed genes (DEGs) trigger by substrate type (plus/ minus lactate) vs growth stage (stationary phase vs growth phase) (**A**), the numbers of DEGs trigger by substrate type vs growth stage (**B**). The overlap of circles was defined as genes affected by both substrate type and growth stage.

As shown in Fig. 4, a total of 295 DEGs in expression pattern were substrate and/or growth dependent, of which 31 genes were substrate (lactate) dependent, 228 genes were growth dependent, and 36 genes were substrate-growth dependent (Fig. 4A). Specifically, 11 and 20 lactate-dependent genes were significantly upregulated and downregulated, as well as 98 and 130 growth-dependent genes were significantly upregulated and downregulated, respectively (Fig. 4B). It was suggested that the differences in gene expression are stronger for stationary phase vs growth phase than for plus/minus lactate. Similar results was observed for *C. thermocellum*, in which growth rate had stronger effects on gene expression than substrate type [25].

### Expression of Glycolysis Genes

**Fig. 5:**
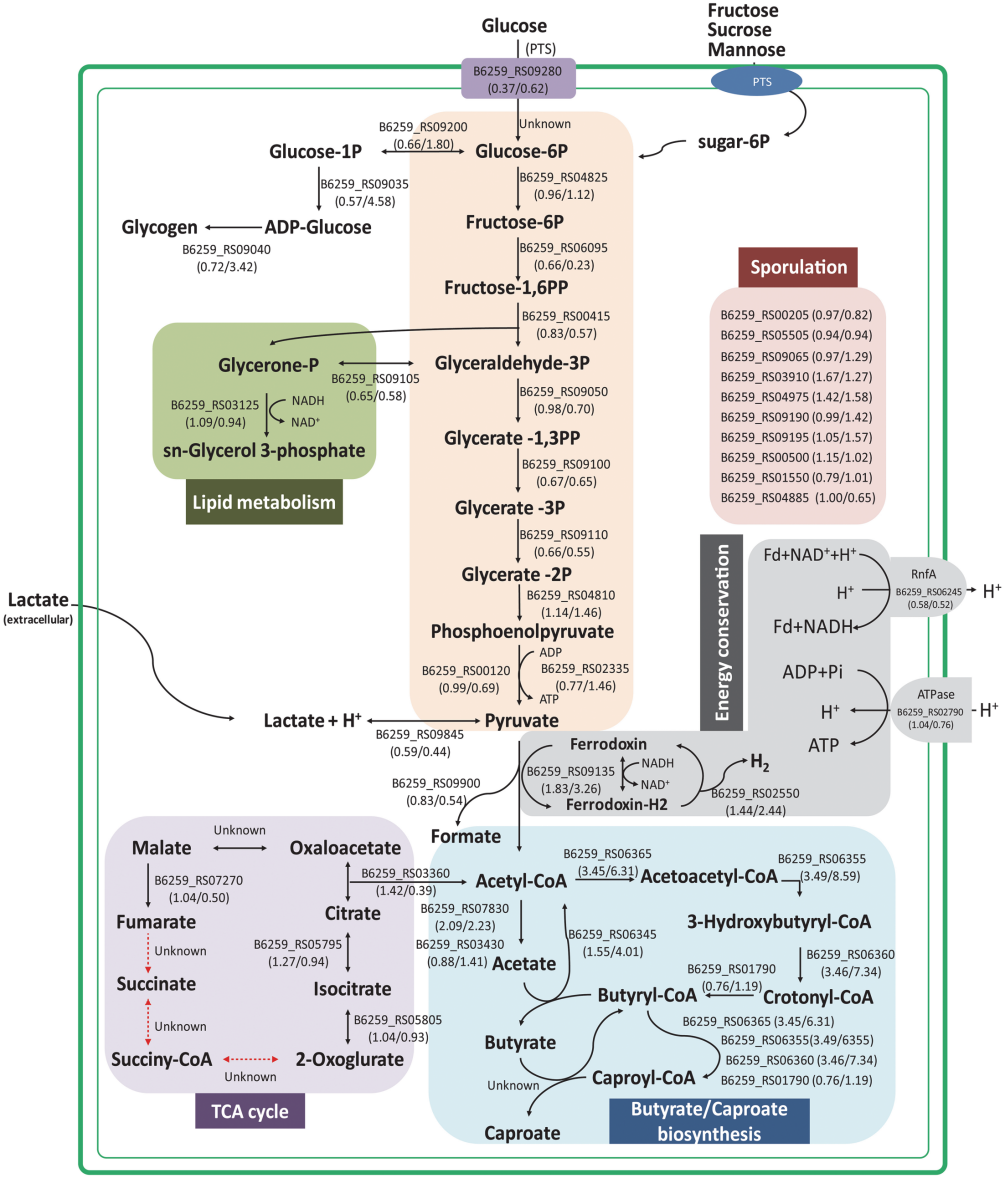
The overview of the central metabolic pathway in strain CPB6 with fold changes (FCs) of the expression of genes. The number in parentheses represents the FC of TPM between the lactate supplemented cells vs. control cells; the first and the second numbers in the same parentheses represent the gene expression FC in the growth and stationary phases, respectively. Black solid lines symbolize enzymatic reactions. Red dashed lines mark enzymatic reactions for which corresponding enzymes are probably not encoded in strain CPB6.

An overview of the metabolic pathway in strain CPB6, and the expression levels of genes involved in key metabolic processes with their fold change (FC) were shown in Fig. 5 and Table 3. Most glycolytic genes were expressed at a relatively high level (TPM>150) between the lactate-supplemented cells and control cells, but there was no significant difference (*p* > 0.05) between them at the growth phase. Three glycolytic genes exhibited different expression patterns at the stationary phase. Gene encoding phosphofructokinase (Pfk, B6259_RS06095) was significantly downregulated (*p* < 0.05), while genes encoding glucose-1-phosphate adenylyltransferase (GlgC, B6259_RS09035) and 1, 4-alpha-glucan branching enzyme (GlgB, B6259_RS09040) were upregulated by 4.58 and 3.42-fold (*p* < 0.05) in the lactate-supplemented cells compared with control cells, respectively. GlgB and GlgC are typically associated with glycogen synthesis, why expression of these genes be affected by lactate supplementation remains unclear. Overall, the addition of lactate has little impact on the expression of glycolytic genes.

Two ldh genes (B6259_RS09845 and RS06770), encoding L-lactate dehydrogenase and D-lactate dehydrogenase, were detected in strain CPB6 transcriptome, respectively. The two ldh genes were expressed at low levels in the lactate-supplemented cells and control cells (Table 3), and there was no significant difference in the expression level between the two groups. The gene encoding pyruvate: ferredoxin (flavodoxin) oxidoreductase (Pfor, B6259_RS09135) was upregulated by 1.83- and 3.26-fold (*p* < 0.05) in the lactate supplemented cells than in control cells during the growth and stationary phases, respectively.

### Expression of Butyrate- and CA-Producing Genes

The enzymes involved in the butyrate formation include acetyl-CoA acetyltransferase (AtoB), 3-hydroxybutyryl-CoA dehydrogenase (Hbd), enoyl-CoA hydratase (Crt), NAD-dependent butyryl-CoA dehydrogenase/Electron transfer flavoprotein complex (Bcd/Etf complex) and butyryl-CoA: acetate CoA transferase (CoAT) [7, 16]. In the present study, genes encoding AtoB (B6259_RS06365), Crt (B6259_RS06360) and Hbd (B6259_RS06355) maintained at very high expression levels (TPM>3000) in the lactate-supplemented cells, and were upregulated by 3.5-8.6 folds (*p* < 0.05) compared with control cells without lactate. Bcd (B6259_RS01790) was expressed at a very high level in the lactate-supplemented cells and control cells throughout the growth and stationary phases, but there was no difference between two groups. EtfAB (alpha unit, B6259_RS01785 and beta unit, B6259_RS01780) showed the Bcd-like expression profile. Another Bcd (B6259_RS02600) was expressed at relatively low level at the growth phase, but its expression was upregulated 4.5-fold (*p* < 0.05) at the stationary phase. One CoAT gene (B6259_RS06345) showed high expression level in the two cultures (TPM>150), and it was markedly upregulated by 4-fold (*p* < 0.05) in the lactate-supplemented cells than in the control at the stationary phase.

Additionally, the gene encoding phosphate acetyltransferase (Pta, B6259_RS07830) was remarkably upregulated (*p* < 0.05) at the two phases with the addition of lactate (Table 3). The expression of acetate kinase (Ack, B6259_RS03430) showed no change (*p* < 0.05) in response to the addition of lactate. The two genes can produce acetate from acetyl-CoA (sourced from glycolysis or lactate oxidation), contributing to a dynamic equilibrium of acetate in cultures to some extent. By including the production of H_2_ and CO_2_ into the loop, it could provide a whole picture for carbon balance for the substrate utilization and cell biomass production. Unfortunately, the production of H_2_ and CO_2_ was not monitored in this study. In the future studies, this should be taken into consideration for improvement.

### Expression of Putative ABC Transporter and Sporulation Genes

As shown in Table 3 and Fig. 5, sporulation genes showed similar expression patterns in the lactate-supplemented cells and control cells, *e.g.*, *spo0*, *spoIIID*, *spoVAE*, and *spoYtfJ*, were induced to high expression at the growth and stationary phases, while *spoIID*, *spoIIIAD*, *spoIVA*, *spoVAC*, and *spoVAD* were expressed at low or moderate levels.

Notably, most genes for ABC transporter and substrate-binding protein (SBP) were no significant changes (*p* > 0.05) in the two groups at growth phase, except two ABC transporter genes (B6259_RS00445, B6259_RS00450), and one SBP gene (B6259_RS00440) which were upregulated by more than 2-fold (*p* < 0.05, Table 3) with the addition of lactate. However, many of these genes were markedly upregulated at the stationary phase (*p* <0.05). Specially, B6259_RS07905, _RS07910, _RS00320, _RS00325 and B6259_RS07915 were increased over 10-fold (*p* < 0.05) in the lactate-supplemented cells compared with control cells.

In addition, four phosphotransferase system (PTS) transporter genes, including PTS fructose transporter subunit IIC (B6259_RS00095), PTS glucose transporter subunit IIA (B6259_RS09280), PTS β-glucoside transporter subunit IIABC (B6259_RS01415), and PTS mannitol transporter subunit IICBA (B6259_RS00370), were detected in the transcriptome of strain CPB6. Genes encoding PTS fructose and glucose transporters were expressed at high levels under both groups, but the two genes were significantly downregulated (*p* < 0.05) in the lactate-supplemented cells than in control cells at the growth phase, indicating that the two PTS transporters are sensitive to lactate supplementation. Moreover, the three PTS transporter genes (B6259_RS0095, RS01415 and RS00370) and one ferrous iron transporter gene (B6259_RS03880) were upregulated by 2- to 4-fold at stationary phase (*p* <0.05, Table 3).

### RT-qPCR Verification

The fold-changes in expression of 5 genes (Pfor, AtoB, Hbd, Crt, and CoAT) were measured by RT-qPCR with 16S rRNA as reference gene. The five genes were significantly upregulated in the lactate-supplemented cells compared with control cells (Fig. S5). The RT-qPCR data mainly matched the RNA-Seq of 5 selected genes based FC values, which indicated that our RNA-Seq result is accurate and the conclusion from RNA-Seq should be reliable.

## Discussion

Lactate is a major end-product of glycolysis or energy substrate for many anaerobic bacteria such as *Acetobacterium woodii*, *C. botulinum* and *Desulfotomaculum reducens* [26, 27]. The recent studies show that lactate as electron donor can be transformed into CA in either mixed anaerobes [3, 12, 13], or in the pure anaerobic bacterium [14], but the biochemistry of lactate oxidation to CA and underlying regulatory mechanisms are still obscure. Lactate dehydrogenase (LDH) is the key enzyme in lactate production from pyruvate. LDH catalyzes the reaction converts pyruvate to lactate or the reverse reaction that converts lactate to pyruvate coupled to NADH/NAD^+^ redox [28]. Generally, bacteria that grow on lactate as sole energy and carbon source have a serious energetic problem because of the high redox potential of the pyruvate/lactate pair. Recently, a novel mode of lactate metabolism is proposed for strictly anaerobic bacteria [27], in which the LDH/ Etf complex uses flavin-based electron confurcation to drive endergonic lactate oxidation with NAD^+^ as oxidant at the expense of simultaneous exergonic electron flow from reduced ferredoxin. And that, the lactate metabolism in these strictly anaerobic bacteria is negatively regulated by the transcriptional regulator [29]. In this study, the upregulation of LDHs was not observed with the addition of lactate, indicating that lactate supplementation does not trigger increased expression of LDH. Moreover, the L-ldh (B6259_RS09845) heterologously expressed in *Escherichia coli* BL21 (DE3) exhibits high LDH activity of driving endergonic lactate oxidation in the absence of Fd^2−^, and LDH oxidative activity predominates over reductive activity [30]. These results indicate that the lactate metabolism in strain CPB6 is different from other strict anaerobes. It warrants further investigation concerning the detailed regulatory mechanism of lactate oxidation in strain CPB6.

The bioproduction of CA is a well-known chain elongation process from acetate (C2) to butyrate (C4), and then to caproate (C6) via the reverse β-oxidation pathway [6]. The conversion of C2 to C4 is well understood, but little is known about the key enzymes responsible for caproyl-CoA or CA synthesis. Enzymes (*e.g.*, AtoB, Crt, Hbd, Bcd/EtfAB complex and CoAT) responsible for butyrate synthesis via the reverse β-oxidation are assumed to have the function in the formation of caproyl-CoA and CA [7]. However, *C. tyrobutyricum*, which contains these genes, only produce butyric acid instead of CA [31], while *C. kluyveri* and strain CPB6, which contain these genes, can further elongate C4 to C6 [7, 15]. It indicates that there are differences in structure and function between these genes from different organisms. In this study, the three genes (AtoB, Crt, Hbd) responsible for the conversion of acetyl-CoA to crotonyl-CoA were markedly upregulated (*p* < 0.05) throughout the exponential and stationary phases with the addition of lactate. However, the Bcd (B6259_RS02600) and CoAT (B6259_RS06345) genes were only significantly upregulated at the stationary phase. Provided that the rate of CA accumulation was significantly higher during the stationary phase than the growth phase, the two genes are likely involved in the formation of caproyl-CoA and CA, The CoAT is the key enzyme responsible for the last step of the butyrate formation [31]. Theoretically, high-level expression of the CoAT gene should result in the accumulation of butyric acid, but significant accumulation of CA instead of butyric acid was observed in the lactate-supplemented cultures, suggesting that the CoAT prefers to convert caproyl-CoA to caproate than butyryl-CoA to butyrate. This speculate was verified by expression of the CoAT (B6259_RS06345) in *E. coli* BL21 (DE3). This CoAT protein could catalyze the conversion of both butyryl-CoA to butyrate and caproyl-CoA to caproate, but its catalytic efficiency with caproyl-CoA as the substrate was 3.8 times higher than that with butyryl-CoA [32]. Thus, the CoAT is a key gene that determines whether the final product is butyric acid or caproic acid.

Some bacteria develop into highly resistant spores to protect their genome and cell from certain doom when living conditions become intolerable [33]. It ensures bacterial survival under adverse environmental conditions. Sporulation in *Clostridium* spp. is ordinarily not triggered by starvation but by cessation of growth in the presence of excess carbon source or exposure to oxygen [33]. The two most critical factors involved in the shift to solventogenesis, a decrease in external pH and accumulation of acidic fermentation products, are generally assumed to be associated with the initiation of sporulation in *Clostridium* spp., to some extent [34]. Recent studies show that the sporulation events are uncoupled from the induction of solventogenesis in *C. beijerinckii* [35]. In this study, the sporulation genes showed no significant difference between the lactate supplemented cells and control cells, indicating that the sporulation events are not associated with the production of CA in strain CPB6 until the stationary phase. This may be because low concentrations of CA (1,717 mg/l) are not sufficient to initiate sporulation for strain CPB6.

ABC transporters are ubiquitous membrane proteins that couple the transport of diverse substrates across cellular membranes to the hydrolysis of ATP [36]. ABC transporters are generally divided into importers and exporters on the basis of the polarity of solute movement. ABC importers are found mostly in bacteria and are crucial in mediating the uptake of solutes including sugar, metal ions, and vitamins [37]. ABC transporters play important roles in response to lactate stress. High expression of ABC transporter genes may be of benefit to organisms to maintain intercellular homeostasis under lactate stress [38] or increase intracellular ATP concentrations to protect cells against acidic damage in the initial stage of acid stress [39]. Here, nine ABC transporter genes and six SBP genes were markedly upregulated at the stationary phase. Specially, B6259_RS07905, _RS07910, _RS00320, _RS00325, and B6259_RS07915 were increased over 10-fold in the lactate supplemented cells compared to control cells, demonstrating that these genes are associated with the extrusion of CA from the cell, and the maintenance of osmotic homeostasis in cytoplasm [40].

PTS is a multiple-component carbohydrate uptake system that drives specific saccharides across the bacterial inner membrane while simultaneously catalyzing sugar phosphorylation [41]. Five distinct subfamilies of proteins related to PTS have been identified within the glucose superfamily: the lactose family, the glucose family, the β-glucoside family, the mannitol family, and the fructose family [42]. In this study, genes encoding PTS fructose, β-glucoside, and mannitol transporters were all strikingly upregulated in the lactate-supplemented cells than in control cells at the stationary phase, suggesting that these transporters may be involved in the extrusion of intracellular CA in strain CPB6, similar to the role of ABC transporters [43].

In sum, this study showed that lactate supplementation induced earlier CA production, higher CA titer, and productivity. The gene transcriptional profiles based on RNA-Seq demonstrated that supplemented lactate promoted CA production by altering the expression patterns of genes responsible for crucial metabolic pathways. Specifically, 5 genes (AtoB, Hbd, Crt, Bcd/EtfAB, and CoAT) involved in the reverse β-oxidation pathway, 11 genes encoding ABC transporter, 6 SBP genes, and 4 PTS transporter genes showed high correlation with utilization of lactate and CA production. The findings presented herein provide unique insights into the metabolic effects of lactate on CA production at the gene regulation level.

## Supplemental Materials

Supplementary data for this paper are available on-line only at http://jmb.or.kr.

## Figures and Tables

**Table 1:** Summary of RNA-Seq sequencing and data analysis results.

Sample Name	L1			L2			C1			C2		

	a	b	c	a	b	c	a	b	c	a	b	c
Total reads	22982792	18614536	18087962	16881648	16844058	15343814	18684112	19108148	19099082	19909258	23032120	18662552
No. of read mapped	22684858	18390709	17865180	16713319	16606726	15098988	18437261	18893873	18905208	19631686	22572873	18285805
Ratio of reads mapped (%)	98.7	98.8	98.77	99	98.59	98.4	98.68	98.88	98.98	98.61	98.01	97.98
No. of unique reads mapped	22339068	17915954	17559600	16540060	16324768	14862898	18050316	18505697	18508661	19247256	22187272	17856892
No. of genes with detectable expression	1969	1968	1968	1968	1969	1968	1968	1968	1968	1968	1969	1969
Range in expression levels (TPM)	8.3 - 2.7×10^4^	8.0-1.7×10^4^	11.5-1.7×10^4^	3.2-5.3×10^4^	26.4 -3.7×10^4^	6.6 -7.3×10^4^	4.3-2.0×10^4^	3.8-1.8×10^4^	4.1-1.9×10^4^	5.2-2.4×10^4^	10.8-1.8×10^4^	24.5-1.9×10^4^

L1: cell culture with lactate supplementation from the growth phase;

L2: cell culture with lactate supplementation from the stationary phase;

C1: Control culture without lactate supplementation from the growth phase;

C2: Control culture without lactate supplementation from the stationary phase. a and c represented the biological triplicate samples.

**Table 2:** The differentially expressed genes in culture with/without lactate supplementation during the growth phase.

No.	Gene_ID	Gene name	Gene description	TPM		FC (L1/C1)	*P*-value

				C1	L1		
15 upregulated genes (FC ≥ 2.0); all statistically significant (*P* < 0.05)							
1	B6259_RS06365	AtoB	acetyl-CoA C-acetyltransferase	1224	5204	3.45	7.9E-39
2	B6259_RS06360	Crt	enoyl-CoA hydratase	795	3434	3.46	2.4E-33
3	B6259_RS06355	Hbd	3-hydroxybutyryl-CoA dehydrogenase	1418	6306	3.49	2.3E-27
4	B6259_RS07830	Pta	phosphate acetyltransferase	271	666	2.09	4.3E-24
5	B6259_RS00440	-	methionine ABC transporter ATP-binding protein	51	504	5.25	6.2E-18
6	B6259_RS00450	-	metal ABC transporter substrate-binding protein	30	699	5.69	5.9E-15
7	B6259_RS00445	-	ABC transporter permease	27	446	5.17	6.9E-14
8	B6259_RS08190	CysK	cysteine synthase A	390	7426	4.07	6.4E-10
9	B6259_RS08440	-	unknown function	1048	3598	2.33	5.2E-06
10	B6259_RS06010	-	hypothetical protein	21	89	2.52	8.1E-06
11	B6259_RS07140	-	hypothetical protein	154	470	2.17	3.0E-05
12	B6259_RS01720	CadA	cadmium-translocating P-type ATPase	22	66	2.16	3.9E-05
13	B6259_RS06870	-	Hsp20/alpha crystallin family protein	315	1102	2.21	1.6E-04
14	B6259_RS00455	PepT	peptidase T	37	576	2.26	2.0E-04
15	B6259_RS02585	Bdh	butanol dehydrogenase	82	242	2.04	3.1E-04
19 downregulated genes (FC ≤ 0.5); all statistically significant (*P* < 0.05)							
1	B6259_RS08515	-	peptide ABC transporter substrate-binding protein	98	53	0.48	1.7E-23
2	B6259_RS09280	-	PTS glucose transporter subunit IIA	1200	484	0.37	5.5E-23
3	B6259_RS09735	IlvH	acetolactate synthase small subunit	564	302	0.48	9.4E-19
4	B6259_RS06995	-	hypothetical protein	276	43	0.20	2.7E-18
5	B6259_RS08565	-	hypothetical protein	143	79	0.50	2.3E-13
6	B6259_RS07000	-	sugar ABC transporter permease	113	31	0.30	9.2E-13
7	B6259_RS01525	-	unknown function	2683	1010	0.37	8.4E-12
8	B6259_RS03200	-	unknown function	2683	1010	0.37	8.4E-12
9	B6259_RS07010	Tag	glycosylase	144	46	0.34	9.9E-11
10	B6259_RS07005	-	carbohydrate ABC transporter permease	90	33	0.37	1.1E-09
11	B6259_RS01865	-	DUF2520 domain-containing protein	260	85	0.36	6.7E-09
12	B6259_RS01880	PanD	aspartate 1-decarboxylase	444	156	0.37	9.5E-09
13	B6259_RS01870	PanB	3-methyl-2-oxobutanoate hydroxymethyltransferase	314	105	0.37	1.1E-08
14	B6259_RS01875	Panc	pantoate-beta-alanine ligase	350	115	0.37	1.2E-08
15	B6259_RS01760	-	hypothetical protein	820	369	0.44	8.9E-08
16	B6259_RS02315	-	basic amino acid ABC transporter substrate-binding protein	147	79	0.50	1.8E-07
17	B6259_RS00100	FruK	1-phosphofructokinase	1256	276	0.35	1.7E-06
18	B6259_RS00095	-	PTS fructose transporter subunit IIC	1273	372	0.37	1.8E-06
19	B6259_RS00105	-	DeoR/GlpR transcriptional regulator	1304	278	0.36	3.6E-06

L1: lactate-supplemented cells at growth phase

C1: no-lactate-supplemented cells (control) at growth phase

**Table 3:** The differentially expressed genes within the important metabolic pathways in culture with/without lactate supplementation.

Functional description	Gene_ID	TPM of genes from culture with lactate supplementation^a^		TPM of genes from the Control^a^		RNA relative fold change (Treatment/Control)	

		12h	18h	12h	18h	12h	18h
Glycolysis							
PTS-Glc-EIIA, PTS glucose transporter subunit IIA	B6259_RS09280	484	260	1200	517	**0.37** ^c^	0.62
GlgC, glucose-1-phosphate adenylyltransferase	B6259_RS09035	153	1323	236	241	0.57	**4.58** ^b^
GlgB, 1,4-alpha-glucan branching enzyme	B6259_RS09040	194	745	236	201	0.72	**3.42** ^b^
sugar phosphate isomerase/epimerase	B6259_RS06500	181	175	150	233	1.05	0.88
Pgm, phosphoglucomutase	B6259_RS09200	95	189	127	113	0.66	1.80
Gpi, glucose-6-phosphate isomerase	B6259_RS04825	2015	1789	1833	1818	0.96	1.12
Pfk, phosphofructokinase	B6259_RS06095	426	97	580	516	0.66	**0.23** ^c^
Aldo, fructose-bisphosphate aldolase	B6259_RS00415	749	402	800	891	0.83	0.57
Tpi, triose-phosphate isomerase	B6259_RS09105	224	229	315	493	0.65	0.58
GapA, glyceraldehyde phosphate dehydrogenase	B6259_RS09050	5322	4284	4790	7732	0.98	0.70
Pgk, phosphoglycerate kinase	B6259_RS09100	523	524	705	1029	0.67	0.65
GpmI, 2,3-bisphosphoglycerate-independent phosphoglycerate mutase	B6259_RS09110	203	200	284	469	0.66	0.55
Eno, phosphopyruvate hydratase	B6259_RS04810	41	65	30	52	1.14	1.46
PK, pyruvate kinase	B6259_RS02335	254	102	293	228	0.77	1.46
							
Central pyruvate metabolism							
PpdK, pyruvate phosphate dikinase	B6259_RS00120	1301	823	1163	1535	0.99	0.69
Pfor, pyruvate: ferredoxin (flavodoxin) oxidoreductase	B6259_RS09135	4329	4382	2044	1225	1.83	**3.26** ^b^
Pck, phosphoenolpyruvate carboxykinase	B6259_RS09255	368	159	554	1031	0.62	**0.23** ^c^
PflD, formate C-acetyltransferase	B6259_RS09900	98	188	107	471	0.83	0.54
Adh, alcohol dehydrogenase	B6259_RS03100	200	116	163	159	1.07	0.84
							
Incomplete TCA cycle							
Cs, citrate synthase, citrate lyase	B6259_RS03360	936	187	543	642	1.42	**0.39** ^c^
Aco, aconitate hydratase	B6259_RS05795	227	162	153	201	1.27	0.94
Idh, isocitrate dehydrogenase	B6259_RS05805	237	232	197	291	1.04	0.93
Fum, fumarate hydratase	B6259_RS07270	310	186	260	437	1.04	**0.49** ^c^
Pck, phosphoenolpyruvate carboxykinase	B6259_RS09255	368	159	554	1031	0.62	**0.23** ^c^
							
Hydrogen production							
HydE, [FeFe] hydrogenase H-cluster	B6259_RS02550	113	73	174	44	1.44	**2.24** ^b^
HydF, [FeFe] hydrogenase H-cluster	B6259_RS09690	67	40	50	24	1.43	1.17
							
Lactate fermentation pathway							
D-ldh, D-lactate dehydrogenase	B6259_RS06770	76	88	58	108	1.14	0.95
L-ldh, L-lactate dehydrogenase	B6259_RS09845	79	111	119	295	0.59	**0.44** ^c^
							
Acetate fermentation pathway							
Pta, phosphate acetyltransferase	B6259_RS07830	666	697	271	321	**2.09** ^b^	**2.23** ^b^
Ack, acetate kinase	B6259_RS03430	290	297	288	233	0.88	1.41
							
The reverse β-oxidation pathway							
AtoB, acetyl-CoA C-acetyltransferase	B6259_RS06365	5204	9909	1224	1077	**3.45** ^b^	**6.31** ^b^
Hbd, 3-hydroxybutyryl-CoA dehydrogenase	B6259_RS06355	6306	13975	1418	1022	**3.49** ^b^	**8.59** ^b^
Crt, enoyl-CoA hydratase	B6259_RS06360	3434	7348	795	647	**3.46** ^b^	**7.34** ^b^
Bcd1, butyryl-CoA dehydrogenase	B6259_RS01790	3278	3104	3787	3014	0.76	1.19
Bcd2, butyryl-CoA dehydrogenase	B6259_RS02600	42	313	41	66	0.90	**4.49** ^b^
EtfA, electron transfer flavoprotein subunit alpha	B6259_RS01785	2657	2968	3175	2572	0.73	1.31
EtfB, electron transfer flavoprotein subunit beta	B6259_RS01780	3996	4830	4357	4169	0.71	1.31
CoAT, butyryl-CoA: acetate CoA-transferase	B6259_RS06345	521	1497	283	330	1.55	**4.01** ^b^
							
Fructose fermentation pathway							
Ppf, 1-phosphofructokinase	B6259_RS00100	276	2174	1256	239	**0.35** ^c^	**7.33** ^b^
							
Starch and sucrose metabolism							
Pyg, glycogen phosphorylase	B6259_RS00300	90	163	121	103	0.66	1.71
MalQ, 4-alpha-glucanotransferase	B6259_RS07805	53	270	55	61	0.85	**4.34** ^b^
Pgm, Phosphoglucomutase	B6259_RS09200	95	189	127	113	0.66	1.80
							
Energy conservation							
energy-coupling factor transporter ATPase	B6259_RS02790	141	104	117	159	1.04	0.76
electron transport complex protein RnfA	B6259_RS06245	230	162	357	362	0.58	0.52
							
Sporulation							
stage 0 sporulation protein	B6259_RS00205	379	279	233	252	0.97	0.82
stage II sporulation protein D	B6259_RS09065	98	59	96	53	0.97	1.29
stage III sporulation protein AD	B6259_RS03910	126	54	87	26	1.67	1.27
stage IV sporulation protein A	B6259_RS04975	65	30	58	16	1.42	1.58
stage V sporulation protein AC	B6259_RS09190	89	46	77	40	0.99	1.42
stage V sporulation protein AD	B6259_RS09195	69	41	66	34	1.05	1.57
stage V sporulation protein AE	B6259_RS00500	292	226	200	167	1.15	1.02
sporulation transcription factor Spo0A	B6259_RS05505	127	115	83	106	0.94	0.94
sporulation transcriptional regulator SpoIIID	B6259_RS01550	213	188	140	207	0.79	1.01
sporulation protein YtfJ	B6259_RS04885	291	183	145	159	1.00	0.65
							
Transporter genes							
ABC transporter permease	B6259_RS00445	446	274	27	235	**5.17** ^b^	1.27
metal ABC transporter	B6259_RS00450	699	628	30	457	**5.69** ^b^	1.52
ABC transporter permease	B6259_RS02670	296	130	441	387	0.60	**0.40** ^c^
ABC transporter permease	B6259_RS02665	180	96	258	231	0.62	**0.48** ^c^
carbohydrate ABC transporter permease	B6259_RS07005	33	124	90	41	**0.37** ^c^	**3.51** ^b^
carbohydrate ABC transporter permease	B6259_RS07905	71	744	71	40	0.90	**12.71** ^b^
carbohydrate ABC transporter permease	B6259_RS07810	39	229	40	45	0.85	**5.48** ^b^
carbohydrate ABC transporter permease	B6259_RS02030	26	71	16	39	1.35	**2.14** ^b^
sugar ABC transporter permease	B6259_RS07910	82	1175	88	50	0.86	**14.74** ^b^
sugar ABC transporter permease	B6259_RS03335	39	401	26	61	1.30	**5.61** ^b^
sugar ABC transporter permease	B6259_RS07815	36	197	37	49	0.85	**4.34** ^b^
sugar ABC transporter permease	B6259_RS07000	31	135	113	38	**0.30** ^c^	**3.48** ^b^
iron ABC transporter permease	B6259_RS00320	53	1278	77	89	0.62	**10.05** ^b^
ABC transporter ATP-binding protein	B6259_RS00440	504	277	51	239	**5.25** ^b^	1.39
ABC transporter ATP-binding protein	B6259_RS00325	60	2032	94	100	0.58	**11.14** ^b^
ABC transporter ATP-binding protein	B6259_RS08900	153	682	233	214	0.58	**3.13** ^b^
ABC transporter ATP-binding protein	B6259_RS07940	190	40	259	94	0.66	0.42
carbohydrate ABC transporter substrate-binding protein	B6259_RS07915	216	3434	203	103	0.93	**14.51** ^b^
maltose ABC transporter substrate-binding protein	B6259_RS03345	30	501	22	37	1.15	**7.65** ^b^
ABC transporter substrate-binding protein	B6259_RS07820	372	1913	451	344	0.73	**4.63** ^b^
sugar ABC transporter substrate-binding protein	B6259_RS02005	30	93	29	48	0.92	**2.29** ^b^
peptide ABC transporter substrate-binding protein	B6259_RS08515	53	78	98	369	**0.48** ^c^	**0.28** ^c^
peptide ABC transporter substrate-binding protein	B6259_RS02685	1385	819	1442	2222	0.85	**0.50** ^c^
ABC transporter ATP-binding protein	B6259_RS02660	238	119	369	320	0.58	**0.45** ^c^
ABC transporter ATP-binding protein	B6259_RS07940	190	58	259	166	0.66	**0.42** ^c^
PTS fructose transporter subunit IIC	B6259_RS00095	372	2117	1273	485	**0.37** ^c^	**3.87** ^b^
PTS glucose transporter subunit IIA	B6259_RS09280	484	260	1200	517	**0.37** ^c^	0.62
PTS β-glucoside transporter subunit IIABC	B6259_RS01415	81	760	134	141	0.54	**4.70** ^b^
PTS mannitol transporter subunit IICBA	B6259_RS00370	29	89	19	44	1.26	**2.34** ^b^
ferrous iron transport protein B	B6259_RS03880	471	389	531	150	0.81	**2.72** ^b^

^a^Data presented as mean of independent triplicates

^b^Significantly upregulated (FC ≥ 2.0, *p* < 0.05)

^c^Significantly downregulated (FC ≤ 0.5, *p* < 0.05)
