# Supplementary material for: Genome-Wide Transcriptomic Analysis of n-Caproic Acid Production in Ruminococcaceae Bacterium CPB6 with Lactate Supplementation
Source: J Microbiol Biotechnol. 2021 Aug 27;31(11):1533–44. doi: 10.4014/jmb.2107.07009 (PMC9705837; doi:10.4014/jmb.2107.07009)
Supplement: Supplementary file 1 [file jmb-31-11-1533-supple.pdf]

https://doi.org/10.4014/jmb.2107.07009

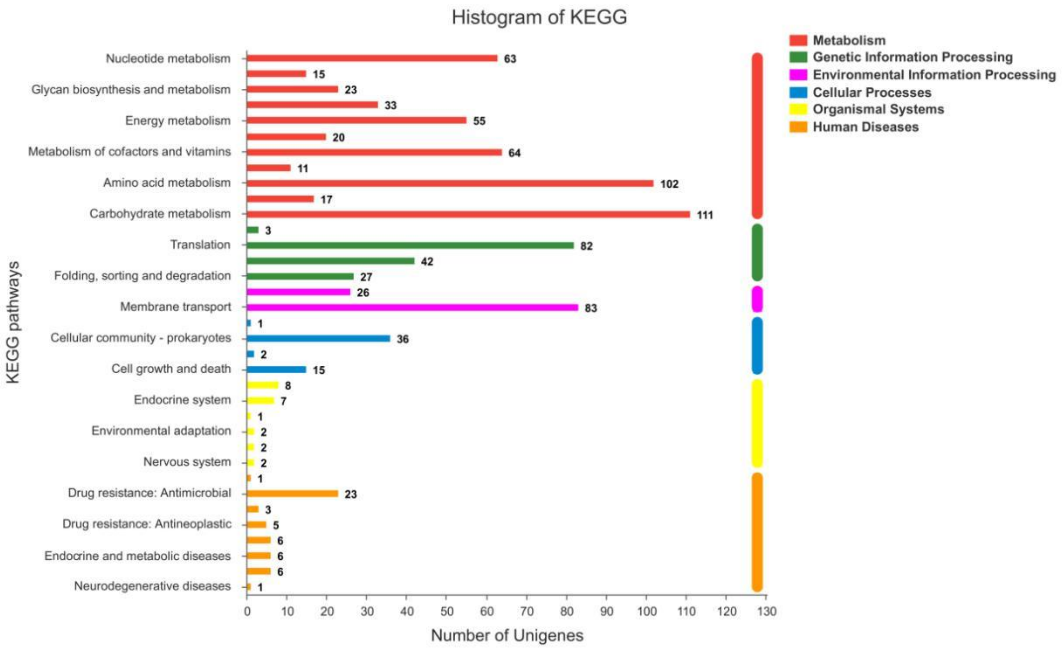

Figure S1. Annotation of genes using Gene Ontology (GO) in the transcriptome of strain CPB6 based on the KEGG pathways. The number of genes within each KEGG pathway was labelled on the right of each bar chart.

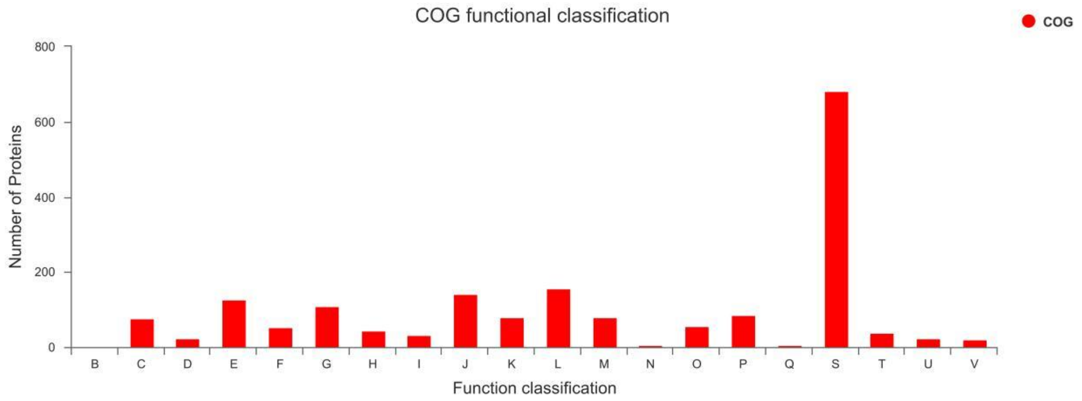

Figure S2. The number of genes within each category based on the Clusters of Orthologous Groups (COGs). B, Chromatin structure and dynamics; C, Energy production and conversion; D, Cell cycle control, cell division, chromosome partitioning; E, Amino acid transport and metabolism; F, Nucleotide transport and metabolism; G, Carbohydrate transport and metabolism; H, Coenzyme transport and metabolism; I, Lipid transport and metabolism; J, Translation, ribosomal structure and biogenesis; K, Transcription, L, Replication, recombination and repair; M, Cell wall/membrane/envelope biogenesis; N, Cell motility; O, Posttranslational modification, protein turnover, chaperones; P, Inorganic ion transport and metabolism; Q, Secondary metabolites biosynthesis, transport and catabolism; S, Function unknown; T, Signal transduction mechanisms; U, Intracellular trafficking, secretion, and vesicular transport; V, Defense mechanisms.

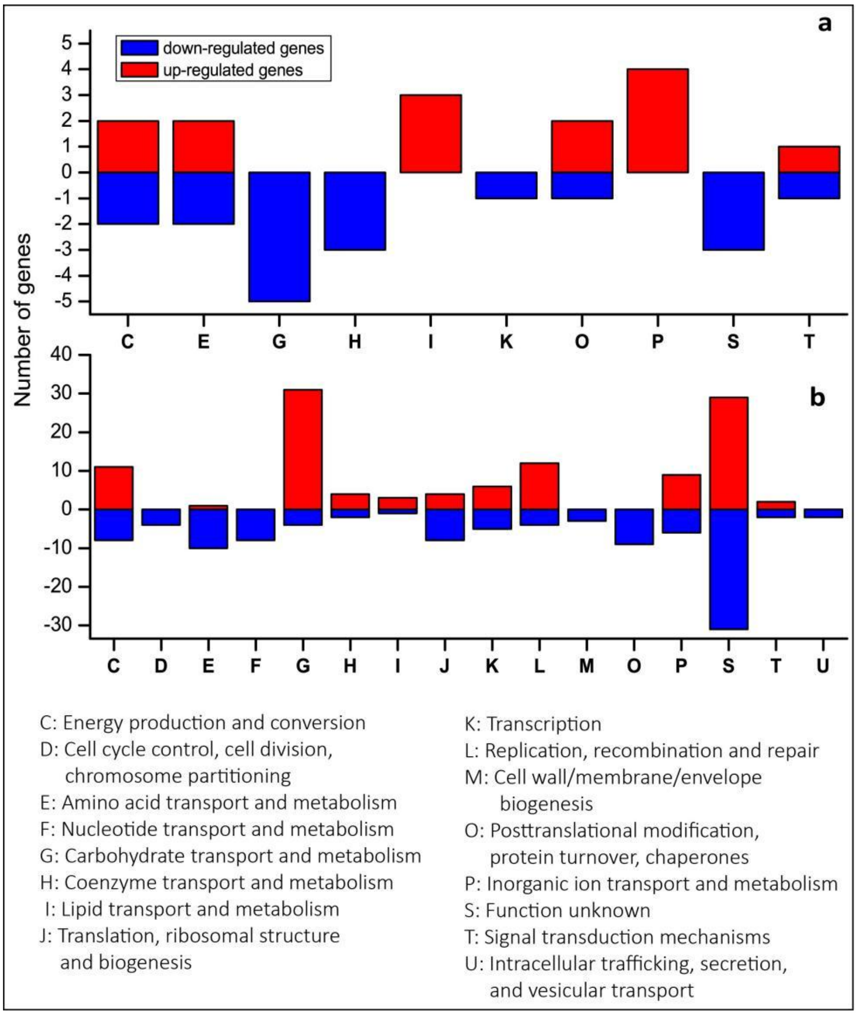

Figure S3. The number of differentially expressed genes (DEGs) between the culture with lactate supplementation and the control falling into each Clusters of Orthologous Groups (COG) categories during the growth phase (a) and the stationary phase (b), respectively. Note that since COG categories overlap, the sum of all COG annotated genes are larger than the total number of all up- and down-regulated genes analyzed.

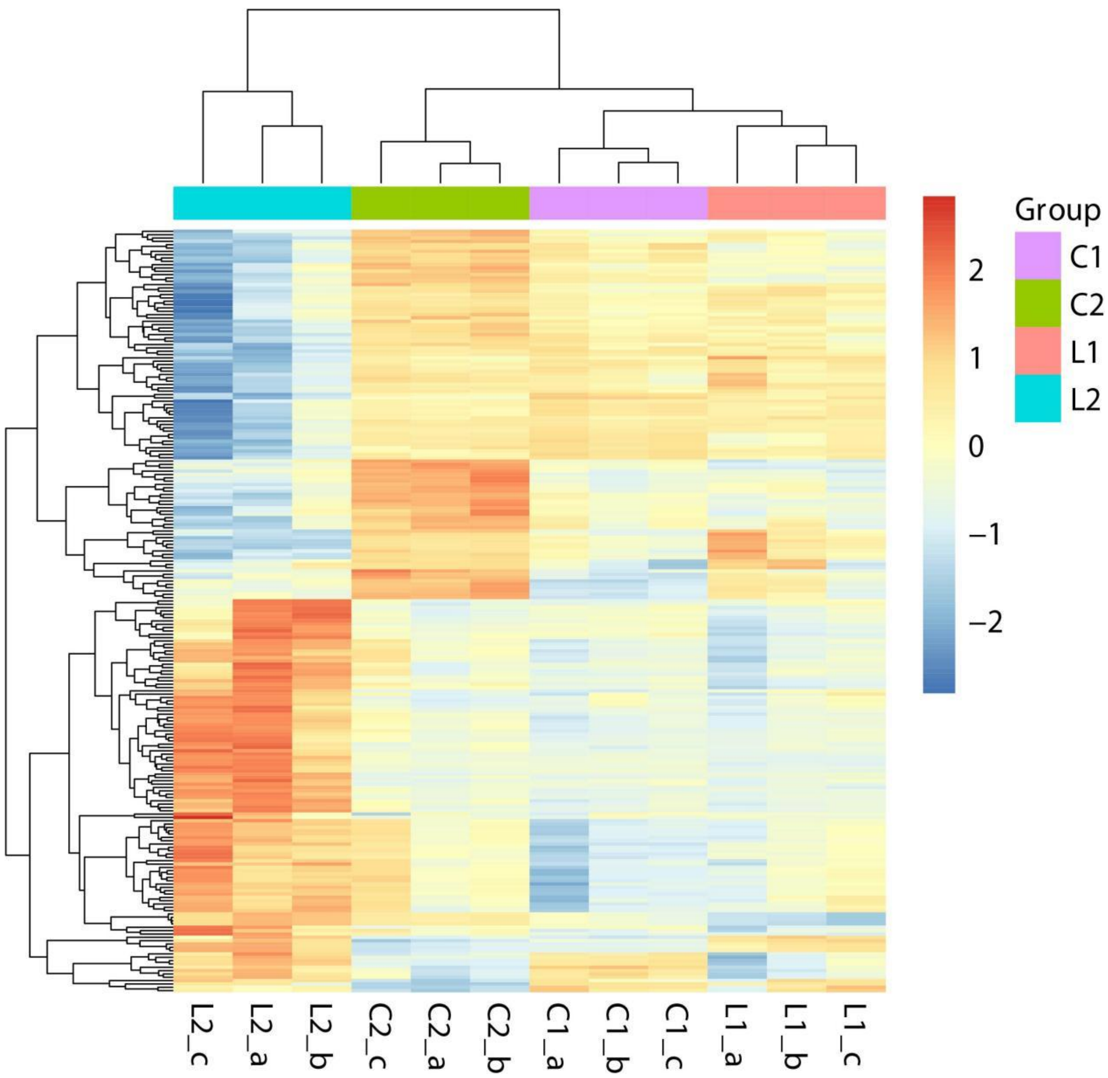

Figure S4. Clustering of differentially expressed genes (DEGs) between the culture with lactate supplementation (L) and the control (C) at the growth and stationary phases. Values of log10TPM were conducted at normalized transformation before clustering. Red indicates genes with high expression, and blue indicates genes with low expression. Each column represented a sample, and each row represented a gene. The left was the tree diagram of clustering gene, and the upper part was the tree diagram of sample clustering, and the bottom was the name of each sample (L: the culture with lactate supplementation, C: the control; Number 1 and 2 represented growth phase and stationary phase, respectively; a, b and c represented the biological triplicate samples).

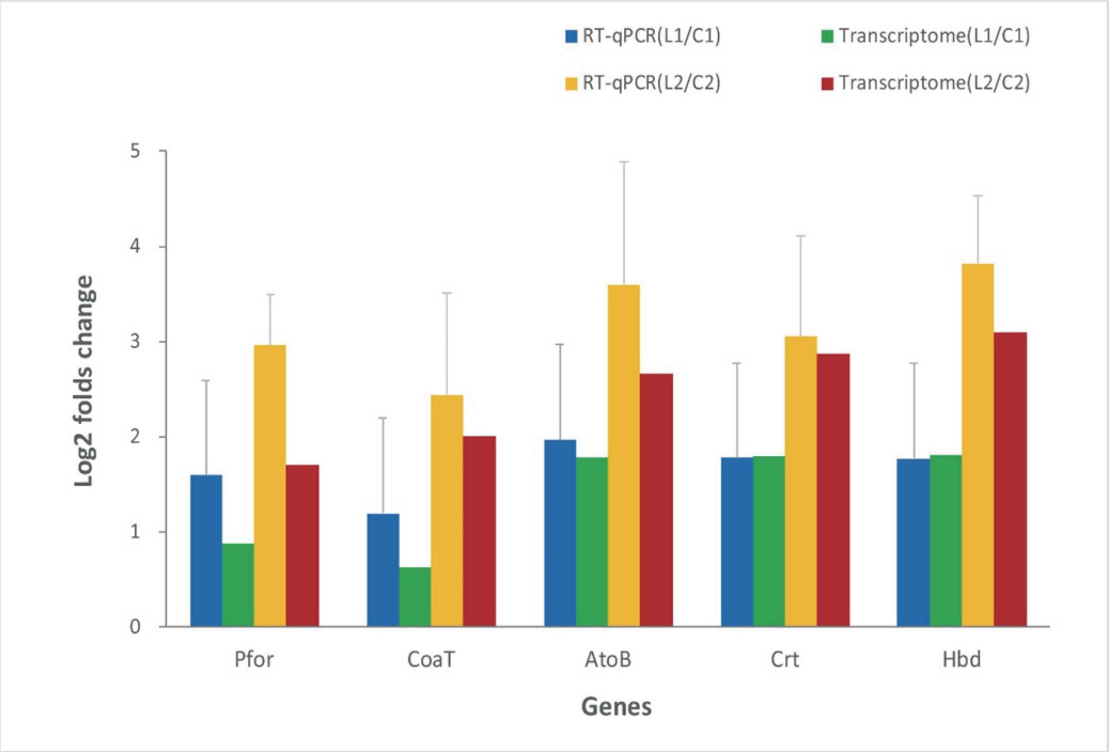

Figure S5. Relative expression of the five gene under different growth culture conditions. C1: Control culture without lactate supplementation from the growth phase; C2: Control culture without lactate supplementation from the stationary phase; L1: cell culture with lactate supplementation from the growth phase; L2: cell culture with lactate supplementation from the stationary phase. cDNA was prepared from three biological replicates, and qPCR was carried out in triplicate.

Table S1 Raw and clean data statistics

Sample Name	Raw reads	Raw Error Rate (%)	Raw Q20 (%)	Raw Q30 (%)	Clean Reads	Clean Error Rate (%)	Clean Q20(%)	Clean Q30(%)
L1_a	2.35E+07	0.014	97.1	93.0	2.30E+07	0.012	98.4	95.1
L1_b	1.90E+07	0.014	96.9	92.5	1.86E+07	0.013	98.2	94.7
L1_c	1.85E+07	0.014	97.1	92.8	1.81E+07	0.012	98.3	94.9
L2_a	1.72E+07	0.014	97.1	92.9	1.69E+07	0.012	98.3	94.9
L2_b	1.72E+07	0.014	97.0	92.8	1.68E+07	0.012	98.4	95.0
L2_c	1.57E+07	0.014	97.0	92.7	1.53E+07	0.012	98.3	94.9
C1_a	1.91E+07	0.014	96.9	92.4	1.87E+07	0.013	98.2	94.7
C1_b	1.95E+07	0.014	97.2	93.1	1.91E+07	0.012	98.4	95.1
C1_c	1.95E+07	0.014	97.1	92.9	1.91E+07	0.012	98.3	95.0
C2_a	2.03E+07	0.014	97.1	92.8	1.99E+07	0.012	98.3	94.9
C2_b	2.35E+07	0.014	97.2	93.2	2.30E+07	0.012	98.4	95.2
C2_c	1.90E+07	0.014	97.2	93.1	1.87E+07	0.012	98.4	95.1

L1: cell culture with lactate supplementation from the growth phase; L2: cell culture with lactate supplementation from the stationary phase; C1: Control culture without lactate supplementation from the growth phase; C2: Control culture without lactate supplementation from the stationary phase. a, b and c represented the biological triplicate samples.

Table S2. The differentially expressed genes in culture with/without lactate supplementation during the stationary phase.

No.	Gene_id	Gene name	TPM[a]		FC	P-value
			C2	L2	(L2/C2)	
	Gene_id	Gene description				
**123 Up-regulated genes (FC ≥ 2.0); all statisticall significant (P < 0.05)**						
1	B6259_RS07910	sugar ABC transporter permease	50.05	1174.52	14.74	8.0E-24
2	B6259_RS03875	ferrous iron transport protein A	452.75	1470.29	3.44	1.9E-23
3	B6259_RS07905	carbohydrate ABC transporter permease	40.28	744.10	12.71	5.6E-22
4	B6259_RS07810	carbohydrate ABC transporter permease	44.90	228.67	5.48	1.3E-20
5	B6259_RS02260	hypothetical protein	213.74	1233.55	5.80	1.8E-20
6	B6259_RS03320	class B sortase	87.10	710.35	7.55	3.5E-20
7	B6259_RS07915	carbohydrate ABC transporter substrate-binding protein	103.13	3434.17	14.51	8.5E-20
9	B6259_RS00320	iron ABC transporter permease	89.20	1277.60	10.05	2.6E-18
11	B6259_RS00325	ABC transporter ATP-binding protein	100.46	2032.40	11.14	4.9E-17
14	B6259_RS07815	sugar ABC transporter permease	49.28	196.87	4.34	3.3E-16
26	B6259_RS07005	carbohydrate ABC transporter permease	40.52	123.81	3.51	2.0E-12
29	B6259_RS03345	maltose ABC transporter substrate-binding protein	37.25	501.15	7.65	8.6E-12
34	B6259_RS03335	sugar ABC transporter permease	60.83	400.66	5.61	5.4E-11
51	B6259_RS07820	ABC transporter substrate-binding protein	343.99	1912.87	4.63	9.5E-09
67	B6259_RS00095	PTS fructose transporter subunit IIC	484.79	2116.74	3.87	1.8E-07
84	B6259_RS07000	sugar ABC transporter permease	37.67	135.34	3.48	1.2E-06
49	B6259_RS02030	carbohydrate ABC transporter permease	38.86	71.08	2.14	3.2E-09
64	B6259_RS02005	sugar ABC transporter substrate-binding protein	47.50	92.83	2.29	8.7E-08
91	B6259_RS08900	ABC transporter ATP-binding protein	214.06	681.91	3.13	2.8E-06

8	B6259_RS00105	DeoR/GlpR transcriptional regulator	217.10	2260.62	8.39	1.0E-18
12	B6259_RS00330	DUF624 domain-containing protein	98.89	1961.68	10.94	1.0E-16
13	B6259_RS03885	FeoB-associated Cys-rich membrane protein	332.50	900.86	2.92	3.1E-16
15	B6259_RS00315	hypothetical protein	153.81	1440.76	7.54	6.4E-16
16	B6259_RS00100	1-phosphofructokinase	239.27	2173.76	7.33	8.4E-16
17	B6259_RS03325	hypothetical protein	155.60	4574.59	10.99	5.6E-15
18	B6259_RS06355	3-hydroxybutyryl-CoA dehydrogenase	1022.29	13975.26	8.59	1.0E-14
19	B6259_RS02520	acetyl-CoA synthetase	47.03	325.31	6.34	1.4E-14
20	B6259_RS07985	XRE family transcriptional regulator	469.11	2126.50	4.41	6.8E-14
21	B6259_RS08545	helix-turn-helix domain-containing protein	70.89	227.74	3.51	1.1E-13
22	B6259_RS02265	DNA alkylation repair protein	170.39	954.34	5.24	1.2E-13
23	B6259_RS02270	cytidylate kinase-like family protein	206.19	1032.65	4.80	1.5E-13
24	B6259_RS05445	MBL fold metallo-hydrolase	74.91	328.39	4.47	5.6E-13
25	B6259_RS06360	enoyl-CoA hydratase	647.43	7347.55	7.34	1.1E-12
27	B6259_RS06995	hypothetical protein	37.68	190.40	5.00	4.9E-12
28	B6259_RS08560	spore coat associated protein CotJA	294.65	922.54	3.31	6.6E-12
30	B6259_RS05440	exodeoxyribonuclease III	129.11	434.58	3.56	1.0E-11
31	B6259_RS02560	ATP-dependent sacrificial sulfur transferase LarE	61.08	209.97	3.54	3.0E-11
32	B6259_RS09030	glucose-1-phosphate adenylyltransferase subunit GlgD	105.61	883.16	6.20	3.5E-11
33	B6259_RS06365	acetyl-CoA C-acetyltransferase	1077.22	9909.02	6.31	4.2E-11
35	B6259_RS00980	ribulokinase	45.52	89.57	2.31	6.2E-11
36	B6259_RS09955	N-acetyltransferase	108.19	238.91	2.52	7.7E-11
37	B6259_RS02595	FprA family A-type flavoprotein	87.28	466.44	4.89	1.9E-10
38	B6259_RS07900	alpha-glucosidase	38.64	145.79	4.00	2.7E-10
39	B6259_RS02570	nickel pincer cofactor biosynthesis protein LarC	81.18	365.00	4.25	2.8E-10
40	B6259_RS02600	acyl-CoA dehydrogenase	66.04	312.69	4.49	4.4E-10
65	B6259_RS06345	butyryl-CoA:acetate CoA-transferase	330.30	1497.47	4.01	8.8E-08
41	B6259_RS00975	hypothetical protein	51.75	93.18	2.11	5.2E-10
42	B6259_RS07805	4-alpha-glucanotransferase	60.67	269.99	4.34	9.2E-10
43	B6259_RS09025	glycogen synthase GlgA	145.38	1415.84	6.04	1.4E-09

44	B6259_RS08550	recombinase family protein	62.53	148.01	2.70	1.5E-09
45	B6259_RS03350	alpha-glycosidase	62.11	787.84	6.50	1.5E-09
46	B6259_RS08555	spore coat protein CotJB	252.33	605.61	2.66	1.8E-09
47	B6259_RS01415	PTS beta-glucoside transporter subunit IIABC	140.53	759.67	4.70	2.2E-09
58	B6259_RS00370	PTS mannitol transporter subunit IICBA	44.43	89.16	2.34	2.6E-08
77	B6259_RS03880	ferrous iron transport protein B	149.84	389.22	2.72	6.2E-07
48	B6259_RS02550	[FeFe] hydrogenase H-cluster radical SAM maturase HydE	89.77	173.84	2.24	2.7E-09
50	B6259_RS07010	glycosylase	53.38	182.17	3.63	6.1E-09
52	B6259_RS09035	glucose-1-phosphate adenylyltransferase	240.73	1322.85	4.58	1.1E-08
53	B6259_RS01825	V-type ATP synthase subunit C	45.41	78.73	2.04	1.7E-08
54	B6259_RS10000	polyribonucleotide nucleotidyltransferase	42.15	72.20	2.01	1.8E-08
55	B6259_RS02145	hypothetical protein	11071.96	71952.9 0	4.80	1.9E-08
56	B6259_RS00335	beta-phosphoglucomutase	60.79	139.33	2.67	2.2E-08
57	B6259_RS01420	alpha%2Calpha-phosphotrehala se	135.12	653.56	4.16	2.6E-08
59	B6259_RS08905	hypothetical protein	279.85	620.18	2.45	2.9E-08
60	B6259_RS00380	PTS mannitol transporter subunit IIA	76.67	157.51	2.35	4.0E-08
61	B6259_RS08535	DUF3298/DUF4163 domain-containing protein	37.48	67.22	2.08	4.3E-08
62	B6259_RS02020	glycoside hydrolase family 65 protein	33.14	65.71	2.32	4.4E-08
63	B6259_RS02090	dihydroxyacetone kinase subunit DhaK	54.47	110.83	2.36	8.2E-08
66	B6259_RS01835	V-type ATP synthase subunit A	41.55	80.08	2.27	1.1E-07
68	B6259_RS02040	beta-phosphoglucomutase	57.59	106.12	2.16	1.9E-07
69	B6259_RS00435	elongation factor G	106.79	537.26	4.16	2.0E-07
70	B6259_RS02095	hypothetical protein	171.01	353.20	2.31	2.2E-07
71	B6259_RS00390	phosphotransferase	37.37	75.54	2.36	3.1E-07
72	B6259_RS00385	HPr family phosphocarrier protein	82.00	170.55	2.32	3.0E-07
73	B6259_RS02035	glycosyl hydrolase	37.95	78.89	2.43	3.8E-07
74	B6259_RS07895	hypothetical protein	103.40	197.33	2.14	4.1E-07
75	B6259_RS02085	dihydroxyacetone kinase subunit L	66.34	137.27	2.38	5.7E-07
76	B6259_RS07800	glycogen/starch/alpha-glucan	88.52	429.62	4.06	5.8E-07

		family phosphorylase				
78	B6259_RS01425	trehalose operon repressor	166.40	608.51	3.35	6.3E-07
79	B6259_RS07510	DNA polymerase IV	131.22	384.83	3.01	6.6E-07
80	B6259_RS00400	L-sorbose 1-phosphate reductase	44.35	85.94	2.25	6.6E-07
81	B6259_RS03615	bacterio-opsin activator	111.19	216.14	2.22	8.4E-07
82	B6259_RS09040	1%2C4-alpha-glucan branching protein GlgB	200.81	744.75	3.42	8.9E-07
83	B6259_RS02080	PTS-dependent dihydroxyacetone kinase	126.06	261.56	2.34	1.1E-06
85	B6259_RS08895	hypothetical protein	217.58	690.93	3.13	1.2E-06
86	B6259_RS01840	V-type ATP synthase subunit B	52.94	97.39	2.17	1.4E-06
87	B6259_RS02535	hypothetical protein	63.66	125.66	2.18	1.5E-06
88	B6259_RS02625	type III pantothenate kinase	91.77	207.22	2.46	1.6E-06
89	B6259_RS01000	hypothetical protein	73.47	131.85	2.04	2.4E-06
90	B6259_RS02115	aquaporin family protein	46.23	84.34	2.10	2.7E-06
92	B6259_RS03340	sugar ABC transporter permease	42.15	512.66	4.59	3.2E-06
93	B6259_RS09135	pyruvate:ferredoxin (flavodoxin) oxidoreductase	1225.35	4382.24	3.26	3.8E-06
94	B6259_RS01845	V-type ATP synthase subunit D	72.69	132.34	2.15	4.8E-06
95	B6259_RS00375	PRD domain-containing protein	49.91	88.64	2.07	6.4E-06
96	B6259_RS01005	transcriptional regulator	51.32	93.93	2.09	1.1E-05
97	B6259_RS02555	hypothetical protein	316.55	1842.56	3.75	1.3E-05
98	B6259_RS02620	ECF transporter S component	152.44	331.70	2.34	1.5E-05
99	B6259_RS03625	DUF2800 domain-containing protein	105.01	289.14	2.79	2.7E-05
100	B6259_RS02565	AIR carboxylase family protein	70.83	345.66	3.48	2.8E-05
101	B6259_RS03695	terminase	118.75	210.26	2.04	4.5E-05
102	B6259_RS02110	glycerol kinase	61.99	116.91	2.14	8.1E-05
103	B6259_RS03690	HNH endonuclease	174.54	310.11	2.02	8.3E-05
104	B6259_RS01760	L-lactate permease	370.92	1134.86	2.79	1.8E-04
105	B6259_RS08565	hypothetical protein	129.20	313.39	2.44	1.8E-04
106	B6259_RS07860	carbon starvation protein A	401.72	1384.12	2.89	2.3E-04
107	B6259_RS08890	cobalamin biosynthesis protein CbiM	187.23	464.85	2.46	2.7E-04
108	B6259_RS06320	ECF transporter S component	583.53	1290.35	2.25	3.2E-04
109	B6259_RS03640	hypothetical protein	68.85	129.62	2.10	3.6E-04
110	B6259_RS01880	aspartate 1-decarboxylase	961.58	1887.70	2.08	5.3E-04
111	B6259_RS07830	phosphate acetyltransferase	321.41	697.23	2.23	7.4E-04

112	B6259_RS03330	LacI family transcriptional regulator	224.79	468.23	2.21	8.9E-04
113	B6259_RS03635	hypothetical protein	307.61	1138.91	2.76	9.5E-04
114	B6259_RS01875	pantoate--beta-alanine ligase	874.21	1789.45	2.13	1.4E-03
115	B6259_RS01865	DUF2520 domain-containing protein	651.06	1311.63	2.11	1.5E-03
116	B6259_RS03630	DUF2815 domain-containing protein	139.90	482.23	2.65	1.7E-03
117	B6259_RS01870	3-methyl-2-oxobutanoate hydroxymethyltransferase	794.85	1533.72	2.02	2.7E-03
118	B6259_RS07350	ribosome-associated translation inhibitor RaiA	3299.88	7621.03	2.23	2.7E-03
119	B6259_RS03620	DNA ligase	211.31	585.69	2.41	3.0E-03
120	B6259_RS07505	4Fe-4S dicluster domain-containing protein	411.53	857.03	2.13	3.7E-03
**122 Down-regulated genes (FC ≤ 0.5); all statisticall significant (P < 0.05)**						
1	B6259_RS06095	6-phosphofructokinase	515.89	96.76	0.23	1.9E-22
2	B6259_RS07165	hypothetical protein	271.34	67.40	0.29	3.0E-20
3	B6259_RS00555	nucleotide exchange factor GrpE	1037.18	176.68	0.22	1.2E-19
4	B6259_RS07070	aminotransferase	315.25	45.59	0.19	3.3E-19
5	B6259_RS04045	diaminopimelate epimerase	320.77	90.60	0.33	3.4E-18
6	B6259_RS07135	DUF134 domain-containing protein	475.47	107.31	0.28	1.5E-17
7	B6259_RS09915	hypothetical protein	532.42	161.13	0.35	1.7E-17
8	B6259_RS00550	heat-inducible transcription repressor HrcA	514.91	86.41	0.22	3.7E-17
9	B6259_RS09910	LL-diaminopimelate aminotransferase	381.87	77.98	0.25	1.5E-16
10	B6259_RS04055	carbamoyl phosphate synthase small subunit	262.98	60.38	0.27	8.6E-16
11	B6259_RS02680	hypothetical protein	1403.18	357.72	0.30	1.4E-15
12	B6259_RS08270	DegV family protein	485.30	106.93	0.27	7.1E-15
13	B6259_RS06870	Hsp20/alpha crystallin family protein	1751.70	217.00	0.18	1.0E-14
14	B6259_RS08370	16S rRNA (guanine(966)-N(2))-methyltransferase RsmD	201.29	69.94	0.40	1.8E-14
15	B6259_RS07155	hypothetical protein	267.88	71.24	0.31	1.1E-13
16	B6259_RS07145	transposase	349.44	90.11	0.31	1.2E-13
17	B6259_RS07130	dinitrogenase iron-molybdenum cofactor biosynthesis protein	377.06	116.62	0.37	2.3E-13
18	B6259_RS00845	hypothetical protein	366.85	120.21	0.38	3.6E-13

19	B6259_RS06940	molecular chaperone HtpG	1169.45	158.48	0.20	9.1E-13
20	B6259_RS03050	hypothetical protein	2604.67	407.88	0.22	2.2E-12
21	B6259_RS06215	NusG domain II-containing protein	169.98	59.72	0.41	2.2E-12
22	B6259_RS08660	dipeptide epimerase	140.36	54.20	0.45	2.7E-12
23	B6259_RS07340	co-chaperone GroES	4354.69	690.44	0.22	2.7E-12
24	B6259_RS03450	ACT domain-containing protein	722.23	249.90	0.40	2.9E-12
25	B6259_RS07140	hypothetical protein	877.17	326.82	0.43	1.8E-11
26	B6259_RS08260	N-acetyltransferase	376.99	129.76	0.41	2.0E-11
27	B6259_RS07125	ATPase	180.94	61.47	0.40	5.5E-11
28	B6259_RS08515	peptide ABC transporter substrate-binding protein	368.77	78.00	0.28	6.2E-11
29	B6259_RS00130	ribose-phosphate pyrophosphokinase	194.21	73.01	0.44	1.9E-10
30	B6259_RS06685	hypothetical protein	171.72	59.93	0.43	2.8E-10
31	B6259_RS00560	molecular chaperone DnaK	1326.89	218.18	0.24	2.9E-10
32	B6259_RS04065	phosphoribosylaminoimidazole succinocarboxamide synthase	260.53	87.28	0.39	1.8E-09
33	B6259_RS09255	phosphoenolpyruvate carboxykinase (GTP)	1030.50	159.25	0.23	2.8E-09
34	B6259_RS06210	FAD:protein FMN transferase	189.13	78.61	0.49	3.4E-09
35	B6259_RS02835	-	1088.67	323.84	0.37	5.4E-09
36	B6259_RS07950	MarR family transcriptional regulator	182.02	75.47	0.47	5.7E-09
37	B6259_RS08365	pantetheine-phosphate adenylyltransferase	391.83	134.30	0.40	9.7E-09
38	B6259_RS04050	LL-diaminopimelate aminotransferase	283.67	97.78	0.41	1.2E-08
40	B6259_RS09735	acetolactate synthase small subunit	917.17	214.94	0.30	1.8E-08
41	B6259_RS03420	hypothetical protein	1063.56	281.28	0.33	3.5E-08
42	B6259_RS10220	ribonuclease P protein component	840.41	252.74	0.36	3.7E-08
43	B6259_RS09845	L-lactate dehydrogenase	295.06	110.52	0.44	4.7E-08
44	B6259_RS06690	hypothetical protein	290.86	118.65	0.48	5.6E-08
45	B6259_RS08605	isopeptide-forming domain-containing fimbrial protein	181.33	75.77	0.50	5.9E-08
46	B6259_RS07120	4Fe-4S dicluster domain-containing protein	434.45	120.99	0.36	6.6E-08
47	B6259_RS06035	ferrous iron transport protein A	8239.42	2008.24	0.31	6.7E-08
48	B6259_RS00565	molecular chaperone DnaJ	417.96	123.58	0.38	7.8E-08

49	B6259_RS07175	type II toxin-antitoxin system Phd/YefM family antitoxin	715.67	256.14	0.43	1.0E-07
50	B6259_RS02670	ABC transporter permease	387.24	129.91	0.40	1.7E-07
62	B6259_RS02665	ABC transporter permease	230.91	96.50	0.48	1.2E-06
96	B6259_RS02660	ABC transporter ATP-binding protein	320.44	119.38	0.45	7.4E-05
121	B6259_RS02685	peptide ABC transporter substrate-binding protein	2222.06	818.78	0.50	8.1E-03
39	B6259_RS07940	ABC transporter ATP-binding protein	165.76	57.61	0.42	1.6E-08
52	B6259_RS02925	adenine phosphoribosyltransferase	464.04	180.50	0.46	2.7E-07
53	B6259_RS09725	DUF1015 domain-containing protein	338.40	107.87	0.39	3.0E-07
54	B6259_RS03545	WYL domain-containing protein	94.20	40.17	0.49	3.1E-07
55	B6259_RS10095	DNA-binding protein	677.71	276.01	0.46	4.5E-07
56	B6259_RS04905	DUF370 domain-containing protein	640.99	243.89	0.45	4.6E-07
57	B6259_RS03445	PFL family protein	249.70	84.75	0.41	5.2E-07
58	B6259_RS07270	fumarate hydratase	437.37	185.71	0.50	6.4E-07
59	B6259_RS06230	Na+-transporting NADH:ubiquinone oxidoreductase subunit D	271.51	103.99	0.45	6.9E-07
60	B6259_RS04060	carbamoyl-phosphate synthase large subunit	238.12	65.10	0.34	7.6E-07
61	B6259_RS05725	flavodoxin family protein	171.61	73.24	0.48	1.0E-06
63	B6259_RS07575	ATP-dependent chaperone ClpB	275.97	76.27	0.37	1.3E-06
64	B6259_RS02415	methionine adenosyltransferase	245.35	87.42	0.44	1.7E-06
65	B6259_RS07115	dinitrogenase iron-molybdenum cofactor	1181.02	343.40	0.38	1.8E-06
66	B6259_RS07415	nucleoside kinase	340.76	121.62	0.44	2.0E-06
67	B6259_RS02855	radical SAM protein	259.25	95.21	0.45	2.1E-06
68	B6259_RS09730	acetolactate synthase%2C large subunit%2C biosynthetic type	468.19	126.39	0.35	2.9E-06
69	B6259_RS00850	PTS fructose transporter subunit IIBC	231.70	91.87	0.47	3.0E-06
70	B6259_RS02140	DUF1292 domain-containing protein	2036.15	603.74	0.37	4.5E-06
71	B6259_RS00875	hypothetical protein	584.20	250.71	0.49	5.1E-06
72	B6259_RS07335	chaperonin GroEL	2411.23	458.40	0.31	5.7E-06

73	B6259_RS07875	radical SAM protein	2525.99	409.92	0.28	5.8E-06
74	B6259_RS00360	hypothetical protein	746.07	219.50	0.38	6.3E-06
75	B6259_RS07425	MBL fold metallo-hydrolase	365.71	138.86	0.46	6.3E-06
76	B6259_RS00855	HPr family phosphocarrier protein	2722.69	932.60	0.42	6.6E-06
77	B6259_RS06225	electron transport complex subunit RsxC	243.47	94.48	0.46	6.7E-06
78	B6259_RS05135	-	3272.24	1075.06	0.39	7.0E-06
79	B6259_RS07420	coproporphyrinogen dehydrogenase HemZ	223.95	86.24	0.47	7.0E-06
80	B6259_RS09205	hypothetical protein	2515.66	1023.39	0.49	1.1E-05
81	B6259_RS03600	DNA-binding protein	4906.12	1894.00	0.47	1.3E-05
82	B6259_RS00840	PRD domain-containing protein	307.23	104.98	0.43	1.3E-05
83	B6259_RS04070	amidophosphoribosyltransferas e	281.23	104.12	0.44	1.4E-05
84	B6259_RS02675	hypothetical protein	1140.46	474.00	0.47	1.4E-05
85	B6259_RS02440	UDP-N-acetylmuramate--L-ala nine ligase	259.52	105.22	0.49	1.8E-05
86	B6259_RS00860	FadR family transcriptional regulator	412.91	168.37	0.49	2.2E-05
87	B6259_RS05115	-	655.16	236.93	0.43	2.4E-05
88	B6259_RS06235	FMN-binding protein	492.22	208.78	0.50	2.5E-05
89	B6259_RS03040	AbrB/MazE/SpoVT family DNA-binding domain-containing protein	3874.84	1206.87	0.39	2.5E-05
90	B6259_RS03360	citrate/2-methylcitrate synthase	641.95	187.07	0.39	2.6E-05
91	B6259_RS00355	GGGtGRT protein	995.31	278.92	0.38	3.2E-05
92	B6259_RS07485	inorganic phosphate transporter	349.68	131.59	0.46	5.4E-05
93	B6259_RS09740	ketol-acid reductoisomerase	2189.64	463.83	0.32	6.2E-05
94	B6259_RS10225	50S ribosomal protein L34	10764.23	3154.60	0.38	6.3E-05
95	B6259_RS04265	Crp/Fnr family transcriptional regulator	432.45	179.17	0.50	7.1E-05
97	B6259_RS04030	UDP-N-acetylglucosamine 1-carboxyvinyltransferase	490.74	169.56	0.44	1.0E-04
98	B6259_RS07480	DUF47 domain-containing protein	723.75	233.02	0.41	1.0E-04
99	B6259_RS08265	phosphoenolpyruvate--protein phosphotransferase	298.48	122.24	0.50	1.5E-04
100	B6259_RS03605	hypothetical protein	1044.28	349.86	0.45	1.7E-04
101	B6259_RS02655	oligopeptide ABC transporter ATP-binding protein OppF	309.83	122.06	0.48	2.2E-04

102	B6259_RS02165	3-phosphoserine/phosphohydroxythreonine transaminase	371.57	133.18	0.45	2.6E-04
103	B6259_RS00865	S-methyl-5-thioribose-1-phosphate isomerase	518.37	200.54	0.48	2.6E-04
104	B6259_RS05855	Asp-tRNA(Asn)/Glu-tRNA(Gln) amidotransferase subunit GatA	281.47	110.33	0.48	4.6E-04
105	B6259_RS06030	ferrous iron transport protein A	5861.87	2010.35	0.42	5.2E-04
106	B6259_RS02170	3-phosphoglycerate dehydrogenase	627.55	205.87	0.43	5.3E-04
107	B6259_RS08820	30S ribosomal protein S10	2376.58	854.38	0.44	5.7E-04
108	B6259_RS05430	YebC/PmpR family DNA-binding transcriptional regulator	453.44	185.23	0.49	8.5E-04
109	B6259_RS05450	hypothetical protein	393.33	136.88	0.46	8.7E-04
110	B6259_RS00510	30S ribosomal protein S20	5392.65	1589.39	0.41	1.2E-03
111	B6259_RS07395	HU family DNA-binding protein	1767.72	746.44	0.49	1.3E-03
112	B6259_RS07370	septum formation initiator family protein	1992.23	754.97	0.48	1.4E-03
113	B6259_RS07960	hypothetical protein	20211.30	5842.73	0.41	1.5E-03
114	B6259_RS06780	hypothetical protein	3526.43	1203.15	0.45	2.1E-03
115	B6259_RS08815	50S ribosomal protein L3	845.83	325.98	0.48	3.2E-03
116	B6259_RS05865	aspartate--tRNA ligase	378.91	149.79	0.50	3.4E-03
117	B6259_RS01600	ribonuclease Y	764.20	267.09	0.48	3.7E-03
118	B6259_RS08810	50S ribosomal protein L4	1316.19	473.61	0.47	3.7E-03
119	B6259_RS05820	thioredoxin	2836.93	1083.99	0.49	4.9E-03
120	B6259_RS04860	50S ribosomal protein L28	10982.47	3555.02	0.45	5.5E-03
122	B6259_RS06025	ferrous iron transport protein B	2079.90	768.55	0.49	9.8E-03

L2: lactate-supplemented cells at stationary phase; C2: no-lactate-supplemented cells (control) at stationary phase.

Table S3. Primers used in this study.

Primers	Sequence (5′-3′)	Length (bp)	Targeting Gene
16S_F357	CCTACGGGAGGCAGCAG	169	16S rRNA
16S_R518	ATTACCGCGGCTGCTGG		
Hbd-F	CTACTGCTGAGGATATTGATACCG	138	Hbd
Hbd-R	TGGAATCGCCAGTCTCGTT		
Crt-F	TTTGTCGGCATTATCACCATC	100	Crt
Crt-R	CCGCATCAATAGCGTCAAC		
AtoB-F	GCAGGTATCCCGATTAGCAC	157	AtoB
AtoB_R	GCAGATAAGGAGCGTTGGAC		
CoAT_F	ACAGGTTCCGAGCGTCACTA	125	CoAT
CoAT-R	GAAACCTGGCACATTGCTACA		
Pfor_F	GCAAACAAGAACTCCGTCAAGA	174	Pfor
Pfor_R	ATGGCAGGCAACGAAATCA		
